# Health Care Providers’ Perspectives on a Hybrid Outpatient Stroke Telerehabilitation Program: Qualitative Implementation Study

**DOI:** 10.2196/83081

**Published:** 2026-06-15

**Authors:** Marina B Wasilewski, Shaghayegh Mirbaha, Karl Wong, Gary Siu, Siobhan Donaghy, Justin Huynh, Sarmiya Uruthiralingam, Michelle L A Nelson, Elizabeth Linkewich, Ron Lacombe, Matthew Godleski

**Affiliations:** 1 St. John's Rehab, Sunnybrook Research Institute, 285 Cummer Ave, North York, ON, M2M 2G1, Canada, 1 416-226-6780 ext 57957; 2 Department of Occupational Science and Occupational Therapy, Temerty Faculty of Medicine, University of Toronto, Toronto, ON, Canada; 3 Rehabilitation Sciences Institute, Temerty Faculty of Medicine, University of Toronto, Toronto, ON, Canada; 4 Institute of Health Policy, Management and Evaluation, Dalla Lana School of Public Health, University of Toronto, Toronto, ON, Canada; 5 Bruyère Health Research Institute, Ottawa, ON, Canada; 6 University Hospital, London Health Science Centre, London, ON, Canada

**Keywords:** stroke telerehabilitation, virtual care, hybrid care, implementation evaluation, outpatient care

## Abstract

**Background:**

Although patient outcomes are improved by stroke rehabilitation, the suggested amount of therapy is rarely maintained. The COVID-19 pandemic aggravated this situation further due to disruptions in health care. One solution was the rapid and extensive transition to virtual care. A hybrid outpatient stroke telerehabilitation program (HOSTP) was introduced by St John’s Rehab—a tertiary rehabilitation hospital in Toronto, Ontario. The HOSTP integrated in-person and virtual care in an effort to alleviate long-standing obstacles that challenge stroke rehabilitation.

**Objective:**

This study explored health care providers’ (HCPs) experiences with the HOSTP and their perspectives on its implementation, quality, and impact to determine the modifications needed to optimize its delivery and sustainability.

**Methods:**

A qualitative implementation study was conducted, with semistructured interviews conducted among HCPs involved in the HOSTP. The interview guide was informed by the CFIR (Consolidated Framework for Implementation Research). In total, 14 HCPs were recruited and interviewed from St John’s Rehab outpatient program. Interview transcripts were analyzed using a 2-stage analytic approach involving inductive thematic analysis, followed by deductive categorization using CFIR.

**Results:**

Four main themes were identified across CFIR domains: (1) adaptability and flexibility of the hybrid care model (intervention characteristics), (2) alignment with patient needs and resources (outer setting), (3) the impact of organizational resources and infrastructure (inner setting), and (4) variability in provider confidence and perceptions of virtual care (characteristics of individuals). Key determinants were identified as adaptability, patient-related factors, resource availability, and provider beliefs about virtual care.

**Conclusions:**

Our findings suggest that, from HCPs’ viewpoints, optimizing virtual care processes and resources may support access and care quality within hybrid outpatient stroke rehabilitation. HCPs viewed maintaining virtual care as important for supporting ongoing access and patient-centered care. Lastly, optimizing the benefits and mitigating the drawbacks of hybrid care can ensure future integration of virtual care into standard outpatient stroke rehabilitation.

## Introduction

Stroke prevalence has increased by 85% over the past 30 years and continues to be a leading cause of adult disability and rehabilitation needs worldwide [1]. In Canada, approximately 878,000 people are living with the effects of stroke, with nearly 90,000 individuals experiencing a new stroke each year [2]. While advances in stroke care have reduced mortality, the individual and societal burden of morbidity remains significant [3]. Half of the people with stroke experience moderate to severe impairments (eg, hemiplegia, speaking difficulties, or cognitive issues) [4] that require long-term support with daily living [3].

Participation in stroke rehabilitation has been widely demonstrated to reduce stroke recurrence, improve functional outcomes, and optimize quality of life [5]. Despite these benefits, the recommended amount of therapy is often not available or achieved [6], with outpatient stroke rehabilitation being particularly underused [7]. This highlights persistent access barriers and the need for innovative models of care that can mitigate these barriers and enhance access to outpatient rehabilitation [7,8]. The advent of the COVID-19 pandemic has exacerbated the general underuse of stroke rehabilitation due to unprecedented disruptions in health care delivery across settings and jurisdictions [1,9]. Recent studies have demonstrated that fewer rehabilitation-eligible people with stroke were discharged to rehabilitation services throughout the pandemic when compared to prepandemic circumstances [10]. One response to these disruptions was the rapid and widespread pivot to virtual care or “telerehabilitation.”

Although stroke telerehabilitation capacity has gradually increased across Canada from 71 hospitals offering the service in 2009 to 307 offering it in 2022, the pandemic was a period of unprecedented expansion in telerehabilitation provision [11]. Telerehabilitation refers to the remote provision of clinical rehabilitation services using information and communication technologies either synchronously (eg, phone calls and videoconferences) or asynchronously (eg, email) [12]. Telerehabilitation delivered through virtual-only care or hybrid program models, with hybrid care models combining in-person and virtual modalities to support more flexible service delivery, whereby rehabilitation services are provided through a combination of in-person and virtual modalities [13]. Telerehabilitation enables the provision of services to manage functional and psychosocial recovery of people with stroke, with evidence demonstrating it to be as effective as usual in-person care at improving motor function, performance of activities of daily living, independence, and self-efficacy [14-16]. Despite these positive impacts and the important role telerehabilitation can play in reducing disability and improving quality of life [17], its uptake prior to the COVID-19 pandemic was slow, with little focus placed on telerehabilitation delivery or its translation from research into stroke practice [1].

Throughout the pandemic, telerehabilitation often served as the only link to care for people with stroke when outpatient services were suspended or reduced. Although virtual care was mobilized out of necessity during the pandemic, it has the potential to mitigate long-standing resource and geographic barriers that limit the provision of and access to stroke treatment. Notably, telerehabilitation precludes the need for travel, which reduces costs and expands care to people with stroke living in rural communities as well as those facing transportation challenges [18]. In a 2021 survey of over 3000 Canadians living with stroke and other vascular conditions, half wanted the option of virtual care, and 60% rated virtual care as good as in-person. This trend in user perspectives is supported by recent recommendations and best practices for community-based stroke rehabilitation that emphasize both virtual and hybrid care models should be leveraged to provide comprehensive care across the continuum [18,19].

The pandemic has driven enhanced uptake of telerehabilitation, recognition of its efficacy, and provider willingness to integrate it into future practice [18]. Thus, the time is now to work towards maintaining this momentum and translating it into opportunities for improvement and sustainability in order to continue meeting the health care needs of Canadian people with stroke [18]. Although hybrid models may improve access flexibility [1,18], there is limited understanding of how they are integrated into routine outpatient practice, including sustainability, workflow, and provider-level factors. Despite growing adoption of hybrid models of care, there remains limited real-world evidence on how these programs are implemented in practice and what factors impact their sustainability in outpatient stroke rehabilitation settings. By evaluating the implementation of current stroke telerehabilitation initiatives, real-world evidence can be used to optimize and broaden this care model. This includes exploring the complex process of integrating virtual care into usual stroke rehabilitation [1] and understanding for whom stroke telerehabilitation works best and under what circumstances [18,19].

The overarching goal of this study was to evaluate the implementation of an existing hybrid outpatient stroke telerehabilitation program (HOSTP) for people with stroke. Specifically, this study advances knowledge by providing CFIR (Consolidated Framework for Implementation Research)-mapped insights into barriers and facilitators impacting the implementation and sustainability of hybrid outpatient stroke rehabilitation in real-world settings beyond the pandemic era. We aimed to:

1. Explore the experiences of health care providers (HCPs) with the HOSTP and their perspectives on its implementation, quality, and impact.
2. Determine organization, human, and resource modifications that can be made to optimize future iterations of the HOSTP for ongoing delivery.

## Methods

### HOSTP Implementation

The HOSTP is offered through the outpatient program of St John’s Rehab—a tertiary rehabilitation hospital in Toronto, Ontario. From 2015‐2020, St John’s Rehab admitted an average of 2500 patients per year to its outpatient program, with over 300 being people with stroke. In response to the COVID-19 pandemic, the St John’s Rehab outpatient program launched a virtual care initiative to mitigate the service disruptions experienced by community-residing people with stroke. Services that are provided virtually include one-on-one appointments with HCPs, group sessions, and education classes. As the pandemic progressed and some in-person services were restored, this program was adapted into a “hybrid care model.” All patients who participate in the HOSTP undergo, at a minimum, a virtual intake assessment. Modes of care (virtual, in-person, or hybrid) were determined based on clinical judgment and patient needs. Virtual care was primarily delivered using Zoom for Healthcare (Zoom Communications, Inc). After that, patients may receive virtual therapy, in-person therapy, or a combination of both, as needed. The HOSTP serves over 100 people with stroke annually, with patients typically participating in therapy twice a week for 8‐12 weeks. People with stroke receive approximately 16‐24 treatment sessions per care discipline. A total of 30 care providers are involved in the HOSTP, including physiotherapists (PTs), occupational therapists (OTs), PT assistants (PTAs), OT assistants (OTAs), speech language pathologists, social workers, and physicians. HCPs work in various capacities, including clinical, managerial, and administrative roles.

The HOSTP represents an informative implementation case due to its scale (services over 100 people with stroke annually), hybrid design, and duration. It is embedded in a well-established outpatient program at a tertiary rehabilitation hospital, which provides a unique opportunity to evaluate the real-world implementation and impact of this type of hybrid telerehabilitation program.

### Ethical Considerations

This study was approved by the Research Ethics Board at Sunnybrook Health Sciences Center (REB# 5144). To protect confidentiality in this single-site study, all data were deidentified, and roles and identifying details were generalized to minimize identification risk within this single-site setting. Researchers followed up with interested participants, went through the informed consent forms with them, and obtained consent prior to interviews.

### Design

We used a qualitative implementation science approach. Using a qualitative descriptive approach [20], we gained insight into the experiences of HCPs with the HOSTP and explored their perspectives on its implementation, quality, and impact. Qualitative description produces a report that remains near to participants’ own words, making it more meaningful to key stakeholders and easily taken up for the type of actionable change we hope to achieve through this project [20,21]. We also conducted this study through the lens of implementation science by using the CFIR to inform the development of the interview guide and guide the deductive categorization and reporting of findings following an initial inductive thematic analysis [22]. The CFIR outlines 5 domains that have been associated with effective implementation of health care innovations: (1) intervention characteristics (eg, relative advantage and disadvantage of intervention, adaptability and trialability, and intervention complexity), (2) inner setting (eg, networks and communication, implementation climate, and tension for change), (3) outer setting (eg, external policies and incentives, and patient needs), (4) characteristics of individuals (eg, self-efficacy, stakeholders’ knowledge and beliefs about intervention), and (5) implementation process (eg, engaging appropriate individuals, reflecting, and evaluating). The CFIR guided the topics and questions included in our semistructured interview guide and the categorization of thematic data (see Multimedia Appendix 1 for the complete interview guide). We adhered to recognized reporting criteria by applying the Standards for Reporting Qualitative Research (SRQR; Checklist 1) [23] and the Consolidated Criteria for Reporting Qualitative Research (COREQ; Checklist 2) checklists [24]. These reporting guidelines were used to ensure comprehensive reporting of the qualitative study design.

### Participants and Recruitment

HCPs were recruited using purposeful convenience sampling. This sampling technique helped us identify participants with experience directly relevant to the topic of interest while aiming to capture a range of perspectives across disciplines [25]. We purposively sampled for diversity in HCP disciplines (eg, OTs, physiotherapists, OTAs, PTAs, social workers, patient care managers, and service coordinators). To be included in the study, HCPs must have (1) worked full- or part-time at St John’s Rehab outpatient department, and (2) delivered care through the HOSTP at least twice in 6 months prior to recruitment. We recruited participants until data saturation was achieved (ie, no new data emerged in subsequent interviews) [26]. An email requesting participation was sent out to HCPs on the mailing list for the outpatient stroke and neurology unit based on the eligibility criteria. The email described the study’s purpose, benefits/risks of participating, time commitment, and confidentiality and freedom to withdraw at any time.

### Data Collection

One-on-one semistructured interviews were conducted with HCPs using Zoom (Zoom Communications, Inc). The interviews explored participants’ experiences with the HOSTP and were informed by the CFIR. Based on the CFIR, the interview guide included open-ended questions regarding experiences with virtual care (likes and dislikes; characteristics of the individuals), what made virtual care easier/harder, what aspects are going well and can be improved (intervention characteristics), and how virtual care promotes/challenges rehabilitation care in the community (implementation process) [22]. Interviews lasted between 30 and 45 minutes and were audio-recorded, professionally transcribed, and checked for accuracy. Transcripts were used for analysis.

### Data Analysis

We used a 2-stage analytic approach. First, we conducted an inductive thematic analysis following the steps outlined by Braun and Clarke [27], whereby data were deconstructed into isolated fragments followed by reconstruction into overarching themes that described the higher-level messaging in the data pertaining to HCPs’ experiences with the HOSTP. Coding was conducted iteratively, with an initial codebook established and refined through regular team meetings and discussions. Discrepancies in coding were resolved through consensus among the research team. This was followed by a more deductive analysis whereby the experiences of HCPs with the HOSTP were categorized according to the CFIR framework. Three independent researchers (JH, SU, and SM) completed the coding process, and 4 additional researchers (MW, KW, GS, and SD) contributed to the thematic and deductive analyses. Final themes were developed through a collaborative approach among the research team, guided by recurring patterns and relevance to the research objectives.

### Rigor

Analytic rigor was enhanced through multiple strategies. Data saturation was assessed iteratively, with recruitment continuing until no new data emerged. The coding team collaboratively determined that saturation had been reached when repetitive comments were heard from participants and no new information was gathered. Coding was conducted by 3 researchers (SM, JH, and SU) using qualitative analysis software, NVivo (Lumivero), with discrepancies discussed and resolved in team meetings until consensus was achieved. Triangulation between multiple individuals throughout analysis, having regular team meetings, and exercising reflexivity was maintained through ongoing team discussions about the study team’s own biases and experiences that may influence data interpretation. Member checking was not conducted due to budget and time restrictions, which are common challenges for undertaking this practice [28]. Studies reassure, however, that member checking is just one of many valuable techniques (eg, triangulation or reflexivity) that can be used to establish credibility and trustworthiness [29]. An audit trail documenting coding decisions, theme establishment, and analytic processes was kept to ensure that the research process was transparent. Finally, appropriate quotes were provided in the final report to support interpretations of the data, thereby enhancing the reliability of study findings.

## Results

### Overview

We interviewed 14 HCPs, comprised of OTs (n=4), physiotherapists (n=4), social workers (n=1), OTA (n=1), and PTA (n=1), patient care managers who have a professional clinical background in physiotherapy (n=2), and a service coordinator (n=1). Most participants were female (n=12; see Tables1  2 for characteristics and demographic information of HCPs). All 5 CFIR domains were represented in participants’ narratives: intervention characteristics, outer setting, characteristics of the individual, inner setting, and process. Below, we outline the details of each domain, with representative quotes included from HCPs, and the specific CFIR constructs italicized in parentheses (Table 3). We also conducted a complementary thematic analysis to identify cross-cutting themes that extend across these domains, offering a more integrated perspective on the overarching insights from the interviews. These themes represented HCPs’ views on the (1) adaptability and flexibility of the hybrid care model, (2) alignment with patient needs and resources, (3) the role of organizational resources and infrastructure, and (4) variability in provider confidence and perceptions of virtual care. Discussions pertaining to each theme were fairly consistent across participants, with some variation observed across disciplines.

**Table 1.** Characteristics of the health care providers.

Role	Description	Health care providers, n (%)	Years of practice, mean (SD)
Occupational therapist	Uses daily activities to treat the physical, mental, developmental, and emotional conditions that affect a patient’s capacity to carry out day-to-day duties	4 (28.6)	15.75 (7.80)
Physiotherapist	Assess and treat individuals whose movement and function are affected by varied factors, such as aging, conditions, injury, or diseases	4 (28.6)	17.25 (9.21)
Social worker	Works with different stakeholders to identify users’ needs, provide practical and emotional support, as well as assist users and their caregivers to improve their quality of life	1 (7.14)	>25
Occupational/physical therapy assistants	Similar scope of practice as OTs^a^ and PTs^b^, but are only licensed to work under the supervision of registered OTs and PTs	2 (14.29)	10.5 (2.12)
Patient care manager/project manager	Leads a clinical team and is in charge of determining the direction of patient care	2 (14.29)	18 (5.66)
Service coordinator	Complete and oversee a variety of professional assignments to assist and provide services such as casework, treatment, outreach development, and referrals to rehabilitation patients	1 (7.14)	15

a OT: occupational therapist.

b PT: physiotherapist.

**Table 2.** Demographic information of health care providers.

Characteristics	Health care providers (n=14)
Age in years, mean (SD)	43.29 (6.09)
Assigned sex at birth	
Male	3 (21.43)
Female	11 (78.57)
Current gender identity	
Male	3 (21.43)
Female	11 (78.57)

**Table 3.** Health care providers’ (HCPs) insights about the hybrid outpatient telerehabilitation program for people with stroke, categorized by the CFIR (Consolidated Framework for Implementation Research) domain and construct.

CFIR domain and construct/subconstruct	Example
Intervention characteristics	
Adaptability: the degree to which an innovation can be adapted, tailored, refined, or reinvented to meet local needs.	“We are given the flexibility to choose whether we want to see our clients virtually, or we want to be able to see them in person. That definitely makes a big difference, and that definitely has helped us to grow as a team and to be able to provide the best care to our clients.” (HCP09, PCM^a^)
Relative advantage: stakeholders’ perception of the advantage of implementing the intervention versus an alternative solution.	“There is a subset of people who sometimes have trouble getting to [REHAB HOSPITAL], either for geographic reasons or transportation reasons or because they have become ill suddenly and now cannot attend, but maybe they are well enough to do things, just not well enough to come in means me having that option to go virtually means that the care can continue in those situations.” (HCP07, PT^b^) “It is good to see how they do it in their home environment. It’s also good to see how they walk in their home environment because sometimes patients tell you that […] Again, seeing them in their home environment also adds another layer of depth to everything.” (HCP08, PT) “It is a nice feature, especially when we were not letting family members come in. It’s nice that then they can have a family member there to add information or just help them answer some of those questions.” (HCP11, PT) “I think the virtual intake assessments have been a really big win for us both on our side as administrators but also from feedback from our clinicians. It’s given us the opportunity to touch base with a patient a lot sooner, but in a way that the patient doesn’t actually have to come here for things that can be done virtually. So, conversations about what the goals of therapy are, past medical history, all that stuff can be done.” (HCP04, PM^c^) “With discharge planning, I think that’s where you can probably utilize teach-back to really see whether or not someone has been able to meet their goals. You can observe what they're doing, and you can observe them doing the strategies within a different setting, within their home setting. So, I think from a discharge planning perspective, that may be helpful. There may be some pieces with warm handovers.” (HCP03, PT) “It certainly added to the progress of the patient because even if it’s virtual and that patient can’t come in, you still have some amount of therapy, so it’s better than nothing.” (HCP13, OT^d^)
Outer setting	
Patient needs and resources: the extent to which patient needs are accurately known and prioritized by the organization.	“Clients who are fairly independent or have family members that can set them up to be independent, who have a good space in their home, good connectivity, and good equipment on their end, because that can pose problems if they don’t. So, in some ways, it’s more privileged people that benefit most from it.” (HCP02, OT) “As an administrator, when we talk about transitioning someone from our care to the community care, that if we set them up and make them comfortable […]. So, if they’re comfortable accessing virtual care, you’ve eliminated one barrier for patients accessing their own after-rehab programs, and you set them up to be more confident in that way. So, I think it does have a large role in discharge planning.” (HCP04, PM) “I think clients who have experience using video chat, especially Zoom, clients who are fairly independent or have family members that can set them up to be independent, who have a good space in their home, good connectivity, and good equipment on their end, because that can pose problems if they do not. … more privileged people benefit most from it. So those people who are disadvantaged …, they are the people who suffer the most from using virtual care and would benefit more from coming in person, if they can.” (HCP02, OT)
Cosmopolitanism: the degree to which an organization is networked with other external organizations.	“Virtual care can help to facilitate a discharge into the community a little bit better, so it’s not so drastic in terms of you’ve been coming twice a week for 12 weeks, and then all of a sudden it goes from that to, you’re on your own. Maybe there’s a transition period in the final weeks of care where people are more in their home environment.” (HCP04, PM) “So, if we could connect with the community OT or the physio community therapist that’s seeing the patient for a home safety assessment and give our input as well, that would be ideal. The community OT or the community rehab would coordinate care with the outpatient rehab team.” (HCP13, OT)
Inner setting	
Tension for change: the degree to which stakeholders perceive the current situation as intolerable or needing change.	“COVID has definitely accelerated it. It has made it necessary…Sometimes, these things happen, and they force organizations and programs to adapt. And we had to because we had no other choice. We couldn’t shut down our operations. People still were having strokes and amputations and all that stuff. We needed to find a way to be able to see our patients.” (HCP04, PM)
Learning climate: a climate in which leaders express their own fallibility and need for team members’ assistance and input, and team members feel that they are essential, valued, and knowledgeable partners in the change process.	“It was definitely a learning curve just as we were going and just giving advice to each other. We would also have weekly meetings or regular meetings both as an outpatient department [and] as a neuro team.” (HCP08, PT)
Networks and communications: the nature and quality of webs of social networks, and the nature and quality of formal and informal communications within an organization.	“Since we started the whole virtual care intake process. We have working groups here that are looking at all of that constantly. We try to touch base every few months through our neuro team meetings as well as through the working group. So, definitely, our technology has improved.” (HCP14, OT)
Available resources: the level of resources organizational dedicated for implementation and ongoing operations including physical space and time.	“We’ve talked a lot about maybe having an extra external camera so you could have it farther back so that the person on the other end could see your whole body for an exercise demonstration, or at least having a separate screen sometimes so that if you wanted to tilt the screen down to show your feet, you could still see the person’s face at the same time.” (HCP07, PT) “Our Zoom rooms are primarily little, rectangular boxes that were meant as offices and not a Zoom space, and there’s a limited amount that you can do in this space. Unfortunately, we’re a hospital. We’re not going to suddenly find new spaces. So you’ll have to be creative about using space in a way that’s more conducive to therapy.” (HCP05, service coordinator) “We have started a new program or a new part of the program where we do the intake as virtual. And that’s just the first session. That’s to get us more of the past medical history and social history to fill out what we call our neuro database. But then afterwards, they're usually booked for in-person treatment and assessment.” (HCP08, PT) “[There is a document] that guides patients and clinicians through what to expect about virtual care at [REHAB HOSPITAL], including things like how to access Zoom, how to connect with Zoom, how to troubleshoot if they have issues, whom to contact if they have issues, and things like that.” (HCP04, PM)
Compatibility: the degree of tangible fit between the meaning and values attached to the intervention by involved individuals, and how the intervention fits with existing workflows and systems.	“I would say from an OT/PT perspective, the vast majority of clients are coming in for face-to-face intervention. Within the role of speech, there is still, I think, more virtual happening from a speech/language pathology perspective, but OT and PT have transitioned really to the vast majority coming in for treatment.” (HCP01, OT)
Characteristics of Individuals	
Knowledge and beliefs about the intervention: individuals’ attitudes toward and value placed on the innovation, as well as familiarity with facts, truths, and principles related to the innovation.	“My hope is that we’re not really replacing anything that we do in person. Eventually, when things settle down, everything that we do in person will still be in person, but we can leverage virtual care and the infrastructure we already have in place to enhance our program.” (HCP04, PM)
Self-efficacy: individual belief in their own capabilities to execute courses of action to achieve implementation goals.	“When I first started, I didn’t feel very prepared for it. I don’t think anyone really did. It was like, let’s see how this goes so that we could reopen. But the administration and the team were very good at trying to fill in any of those gaps that we didn’t feel comfortable with or troubleshooting a lot of the tech issues surrounding Zoom. It didn't take so long to feel less confident. But in the beginning, it was definitely like, we were all just trying to figure it out.” (HCP11, PT)
Individual stage of change: characterization of the phase an individual is in, as s/he progresses toward skilled, enthusiastic, and sustained use of the innovation.	“I had no clue how it would go, and a long time ago, I said to myself, you have to move with the times. You have to deal with change. You don’t have a choice. I just dealt with it. You have to be adaptable.” (HCP06, social worker)
Process	
Planning: the degree to which a scheme or method of behavior and tasks for implementing an innovation are developed in advance, and the quality of those schemes or methods.	“When we put it together was at the beginning of COVID, so we really didn’t have a clear idea of how we were going to provide virtual care and really what was going to be the scope of virtual care.” (HCP11, PT)
Engaging: attracting and involving appropriate individuals in the implementation and use of the innovation through a combined strategy of social marketing, education, role modeling, training, and other similar activities.	“I think having our clinicians and patients involved both from a planning perspective as well as from an implementation perspective really helped us to a, make sure that there’s engagement in the process and then b, to be able to find solutions that were meaningful both from a patient perspective as well as from a staff perspective.” (HCP03, PT)
Reflecting and evaluating: quantitative and qualitative feedback about the progress and quality of implementation, accompanied by regular personal and team debriefing about progress and experience.	“We launched a virtual care initiative around April and May this year. We’ve had a lot of feedback from staff and patients on what some of the gaps were and what we could do to improve virtual care. And so, we had lots of ideas that related to documentation, consent, how to prepare patients better for virtual care.” (HCP04, PM)

a PCM: patient care manager.

b PT: physiotherapist.

c PM: project manager.

d OT: occupational therapist.

### Domain 1: Intervention Characteristics (Features of the Intervention That Affect Implementation Success)

Participants affirmed that “the hybrid model of care has become normalized in the overall program of outpatients, or at least the neuro program” (HCP07). HCPs spoke of the benefits of having “the flexibility to choose whether we want to see our clients virtually, or we want to be able to see them in person, … has helped us to grow as a team and to be able to provide the best care to our clients” (HCP09; adaptability). HCPs did note, however, that despite the benefits of virtual care, there were challenges and complexities in offering virtual care to the “stroke population as the majority of assessments (ie, balance, gait, and safety) require hands-on contact” (HCP09). Participants were pragmatic in their perspectives on the HOSTP, though, recognizing that “if It is this [virtual care] or nothing, this is definitely better than nothing” (HCP11), as virtual care “certainly adds to the progress of the patient and you still have some amount of therapy” (HCP13; relative advantage).

HCPs felt that the hybrid model of care provided an “opportunity to touch base with patients a lot earlier and quicker” when it came to simple follow-up appointments that “can be done virtually, such as conversations about what the goals of therapy are and past medical history” (HCP04; relative advantage). HCPs also found virtual intake assessments to be especially beneficial in terms of optimizing and accelerating care provision since patient histories were already completed and did not have to be repeated.

Other advantages of virtual care included the ability to increase geographical reach by reducing or eliminating travel to health care facilities, providing an opportunity to “see what [the] patient is doing in their own home environment versus just what they are doing here [outpatient clinic]” (HCP04; relative advantage). Seeing patients in their home environment was especially helpful and provided the unique advantage of being able to witness patients’ organic interactions with “family members that could not come [to the in-person appointment] because they were at work or they could not travel this far” (HCP04), as well as family members’ additional assistance with “providing medical history if there is a memory loss, translating if there is a language barrier, and being prepared to provide hands-on support” (HCP05; relative advantage). This extended to discharge planning, where HCPs explained that doing this virtually allowed them “to see how the patients are functioning at home at discharge” (HCP08).

### Domain 2: Outer Setting (External Impacts on the Intervention Implementation)

HCP comments reflected that the “needs and resources of the patient” was the primary “outer setting” construct that drove their valuation of virtual care. HCPs recognized that the use of virtual modalities for stroke rehabilitation was highly “client-dependent” (HCP02) and that several factors may impact patients’ participation in and benefit from virtual care. Such factors included level of functioning (higher-functioning or lower-functioning), dependency of patients on their caregivers (highly dependent or relatively independent), comfort levels with using technology, and access to technology (needs and resources of the patients).

One area where HCPs felt that virtual care was especially well-aligned with patient needs was during discharge planning. Virtual care was viewed as a way to facilitate reintegration by “providing feedback to the patient in the home environment, as that can be a little bit more of a smooth transition versus a drastic stop to care” (HCP04; needs and resources of the patients). HCPs felt that virtual care allowed them to better “[connect] online and [give] our input to a community OT or the community therapist that’s seeing the patient [who] was referred for a home safety assessment at the same time” (HCP13; cosmopolitanism). These cross-setting communications were viewed as optimizing care integration for patients by creating a “warmer handover to the next episode of care” (HCP03; cosmopolitanism). Thus, the “patient feels comfortable, as well as the person you are sending the patient to now knows that the information, and so they can do the best intervention they can do” (HCP03).

### Domain 3: Inner Setting (Features of the Implementing Institution)

At the onset of the COVID-19 pandemic, there was a strong tension for change given that infection prevention and control regulations resulted in “the entire department [shutting] down within a couple of weeks” (HCP04). HCPs explained that “COVID definitely made [us] think outside the box and still be able to provide the services we need” (HCP14; tension for change). HCPs felt that the pandemic accelerated the uptake of virtual care, along with the realization that different care models and approaches are needed to ensure continuity of care.

As the COVID-19 pandemic progressed, HCPs encountered a learning curve with regard to technology, administration, and care provision, which left them and their department “constantly looking into growing” (HCP09; learning climate). Nevertheless, they felt that team dynamics and workflow were well-established and optimally positioned to deliver a hybrid model of care for the population with stroke. Participants explained that “it is a good communication that exists between the therapist and the management and the client” (HCP09) that leads to a continuum of teamwork towards “refining the model and just looking at how we can use it to our advantage” (HCP10; network and communication). One of the key advantages identified was the fact that virtual care streamlined appointment scheduling since “[HCPs] do not do the setup for arranging the meetings. It is the office that does all of the scheduling” (HCP06) which, in turn, freed up HCPs and allowed them to “focus strictly on providing care for the patient, [since] everything is set up for us” (HCP06; network and communication). Another perceived “win” with regard to virtual care was the virtual intake assessment conducted by the outpatient team. HCPs viewed this as an “opportunity to touch base with a patient a lot sooner...virtually. So, conversations about therapy goals, past medical history, all that can be done.... It is a lot of work for many of our stroke patients to come to the hospital, so we have prevented them from having to come... and it has actually helped direct our assessment a little bit better” (HCP04).

Conversely, HCPs felt like the organization could have been more prepared in terms of having appropriate physical space and equipment set up for virtual care (eg, having cameras external to the ones built into laptops; available resources). One HCP explained that “the physical space where we do our Zoom sessions is a little bit small for some of the physio-type, exercises I might want to demonstrate. It is a combination of the size of the room and [the fact] that right now, we are typically just using a laptop with a little camera on the laptop” (HCP07). In some cases, however, these issues were addressed by the “comprehensive patient-readiness and clinician-readiness documents” (HCP05) designed by the outpatient team (available resources). Further, a few participants suggested the idea of “looking at how we can reconfigure the office rooms to allow therapists to have a little bit more of a conductive environment” (HCP04), as well as “having an external camera or a separate screen so that the person on the other end could see your [provider] whole body for an exercise demonstration” (HCP07).

HCPs’ profession strongly influenced their perception of the virtual components of the hybrid model, with social workers feeling especially positive about virtual care, believing that “the techniques are the same, the counseling is the counseling. I just had to depend more certainly over the phone on the nonverbal [cues, such as] tone of the voice, expressions, and what they say” (HCP06). On the contrary, occupational therapy and physiotherapy practices entail more hands-on care, which led these HCPs to view virtual care as less practical, particularly for neurorehabilitation, compared to in-person care (compatibility). As one HCP reflected:

*I would say from an OT/PT perspective, the vast majority of clients are coming in for face-to-face intervention...OT and PT have transitioned really to the vast majority coming in [in-person] for treatment*.

[HCP01, OT]

### Domain 4: Characteristics of Individuals (Characteristics of the Implementing Individuals)

Although HCPs recognized the importance of hybrid models of care, particularly during the COVID-19 pandemic, for outpatient rehabilitation of people with stroke, they believed virtual care was “an adjunct” (HCP05) to in-person treatment and did not view it as “the only tool” (HCP05; knowledge and beliefs about the intervention). HCPs varied in how confident they were in their capabilities to use the hybrid model of care (self-efficacy). Some were “very tech savvy” (HCP04) and felt more comfortable using virtual modalities, while others felt less comfortable and “did not have any experiences” with novel technologies (HCP04). During the early stages of hybrid care implementation, HCPs reported that most providers and staff “did not have any [or had insignificant] experiences [with] virtual care” and were “trying to adapt to virtual care” by receiving “step-by-step instructions from [other colleagues] learning how to apply technology” (HCP01; individual stage of change). However, over time, the majority of HCPs became more skilled in using technology to provide care (eg, Zoom for Healthcare software).

### Domain 5: Process (Phases of Implementation)

Providers described how the sudden onset of the COVID-19 pandemic affected the outpatient service provisions, resulting in very limited time for detailed planning (planning). As one participant explained, “The entire department shut down within a couple of weeks, and we had to implement an infrastructure for virtual care within those couple of weeks” (HCP04). Although there was a lack of formal evaluation initially when the program began, as the pandemic progressed, both HCPs and patients were involved to a greater extent in the “planning and implementation process” (HCP03; engaging), which ultimately ensured that the outpatient department was “able to find solutions that were meaningful both from a patient perspective and staff perspective” (HCP03). Team debriefing about the progress of the hybrid care model further improved the implementation, as “we have worked on it as a department,” “ran focus groups” (HCP07), and “did some self-reflection to see how we could better incorporate it into the way in which we do what we do” (HCP03; reflecting and evaluating).

## Discussion

### Overview

Our study aimed to explore the implementation of a HOSTP launched at St John’s Rehab. According to the CFIR, for an innovation to be implemented successfully, accounts must be taken of the innovation’s core components and adaptable periphery, the structural and cultural context through which the implementation occurs, the social context within which the organization resides, and an active change process. Our findings highlighted that all 5 CFIR domains were discussed by HCPs as impacting the implementation of the HOSTP. This study extends the telerehabilitation implementation literature by examining how HCP perceptions of and experiences with the HOSTP evolved from its initial pandemic deployment to integration into routine outpatient care. Although the study reflects HCPs’ perspectives within a single HOSTP (rather than objective measures of patient outcomes or system performance), our findings begin to elucidate how perceived flexibility, clinical fit across disciplines, and organizational learning processes influence provider buy-in and sustainability over time.

Several stay-at-home mandates and lockdown orders during the COVID-19 pandemic fast-tracked the adoption of virtual care in Canada [30]. In response to the pandemic, provinces looked enthusiastically to virtual care as a means of safely providing health care services by mitigating the risk of in-person viral transmission. While participants in our study viewed virtual care as being “better than nothing” at the onset of the pandemic, our findings strongly emphasize that it would be difficult to retain buy-in from HCPs for a virtual-only or “virtual first” approach during the postpandemic era. Findings from our study illustrated that HCPs do not view virtual-only models of care positively, especially when the virtual modality does not align with a hands-on approach needed for their clinical role. Other studies have highlighted that this is particularly pronounced among physiotherapists due to the nature of their practice [31], which was echoed by physiotherapists in our study as their therapeutic domains require hands-on physical activities and examinations with their patients. Adding to the literature, our findings suggest that OTs also view certain therapeutic activities within their scope of practice (eg, gait assessment) as requiring in-person care. Conversely, social workers found virtual care to align with their usual clinical tasks.

Across HCP accounts, the hybrid model was viewed as supporting both patient- and family-centered care by increasing flexibility, continuity, and caregiver involvement, while preserving the option for necessary in-person, hands-on rehabilitation. Consistent with Picker principles of person-centered care [32], participants described how virtual components enabled faster access to care, smoother transitions between outpatient and community services, and clearer communication by allowing HCPs to observe patients’ home environments and tailor recommendations accordingly [32-34]. HCPs also perceived that hybrid delivery supported greater involvement and support for family caregivers by enabling them to attend appointments remotely, contribute contextual information, and participate more actively in discharge planning when in-person attendance was challenging [18,35]. Together, these perceived benefits were viewed as supporting a more integrated and responsive model of care that balanced patient preferences, caregiver engagement, and continuity of care, while preserving the need for in-person, hands-on rehabilitation when clinically indicated.

### Recommendations for Future Optimization of Hybrid Models of Stroke Care

Having the flexibility to choose the care provision approach, either through virtual streams or in-person, showcased the potential of the hybrid model of care to be adaptable to the patients’ and settings’ needs (domain 1: intervention characteristics). This echoes findings from previous studies indicating that for a novel care program to be adaptable, modifications of an existing program must fit the organization’s capacity and needs without compromising its integrity and being flexible enough to meet the patient’s needs and settings [36]. The hybrid model of care was positively viewed as a facilitator for community reintegration, aligning well with patient needs during discharge planning. Evidence has indicated that virtual self-management approaches enhance community reintegration, such as socializing and engaging in daily activities that enhance well-being in community-dwelling adults with stroke [37]. Consequently, it is recommended that organizations capitalize on hybrid models of care that enable providers to maintain connections and support people with stroke virtually and in-person once they return to the community (domain 2: outer setting).

Learning environments that involve HCPs and patients need to be fostered as part of the change process to improve knowledge and build skills among virtual care users. Previous studies have demonstrated that in an effective learning environment, the workplace culture is caring and compassionate, leading to the provision of high-quality care and experience for staff, patients, and caregivers [38]. As a result, cultivating supportive learning environments may be important to ensuring that supportive relationships and transformative policies and processes that support innovation and learning are in place (domain 3: inner setting). HCPs in our study reported that virtual care enabled them to work better as a team and to streamline scheduling and communication, which created more time for patient care. This is a key benefit of the virtual aspect of the hybrid care model in as far as it mobilizes Canadian Stroke Best Practice recommendations that virtual hybrid outpatient stroke rehabilitation services should offer case coordination approaches that include regular team communication to discuss assessments, management, goals, and feedback (domain 3: inner setting) [18]. HCPs discussed how upper management involved them in the implementation process and accepted their feedback to iteratively enhance the HOSTP program, which made them feel heard and validated. As a result, ongoing evaluations may help capture the various stages of virtual care implementation and the particular considerations related to each stage (domain 5: process).

Our findings point to several challenges to implementation that may need to be addressed in the future to ensure that hybrid models of outpatient stroke care continue to provide high-quality, patient-centered care. First, choices and alternatives regarding virtual care should be provided to HCPs, and extreme caution should be exercised to avoid “technological determinism,” in which virtual care is considered the best and only mode of care provision [39]. In our study, this was apparent as many HCPs, particularly physiotherapists and OTs, expressed how the relative advantages of virtual care could be diminished for specific professions that require more hands-on assessments and interventions (domain 1: intervention characteristics).

Secondly, HCPs may benefit from receiving training and educational resources that help them both attain and maintain critical competencies needed to provide safe and appropriate virtual care. Despite the rapid adoption of digital tools and technologies in response to the COVID-19 pandemic, most HCPs, patients, families, and caregivers did not receive adequate training on how to use digital tools in health care systems (domain 3: inner setting). In our study, this meant that HCPs had varying levels of self-efficacy in using technology, subsequently leading to very individualized stages of change when it came to adapting to and adopting virtual care (domain 4: individual characteristics).

Thirdly, adequate infrastructure and resources may be important for supporting successful virtual care implementation [40]. Consistent with growing evidence [40,41], our findings suggest that better resourcing and infrastructure in terms of physical space and technological equipment can encourage HCP uptake of this modality and potentially improve the quality of virtual care sessions (domain 3: inner setting). Overall, our findings are consistent with prior research illustrating the acceptance, feasibility, and potential benefits of stroke telerehabilitation [42]. They also align with existing qualitative and implementation studies that identify user perceptions, resource availability, and workflow considerations as key factors influencing implementation [42,43]. In this way, our findings confirm prior evidence regarding the importance of these determinants. However, much of the existing research has emphasized virtual-only implementation during the pandemic, with less attention to the integration and sustainability of hybrid models. Our findings underscore that hybrid models afford flexibility but also ensure that clinical judgment, patient needs, and discipline-specific requirements drive the nature of care delivery.

### Strength and Limitations

A notable strength of this study was our use of a robust theoretical framework to comprehensively evaluate a novel HOSTP that was rapidly deployed during the COVID-19 pandemic [44]. Our combined use of a qualitative approach and implementation science evaluation allowed us to provide rich and in-depth descriptions of HCPs’ experience with the HOSTP while also identifying key implementation successes and areas for improvement. One limitation of our study is that the HOSTP was provided by a rehabilitation hospital in an urban setting, thus potentially limiting the transferability of our findings to programs offered in rural areas (where, eg, issues may arise around technology access, internet reliability, and geographical distance to in-person care). Additionally, this study was conducted at a single site with a relatively small sample size, which may, in turn, limit the transferability of findings to other settings. An additional limitation related to our recruitment approach where participants were recruited using purposeful convenience sampling from a single outpatient unit mailing list. This approach may have introduced selection bias, as those who chose to participate may have been more involved, more available, or held more favorable perspectives toward the program. Therefore, the findings may not fully represent the perspectives of all HCPs, particularly those who may have been less involved or held more critical perspectives. Another limitation relates to the overrepresentation of physiotherapists and OTs in our study. The inclusion of additional health professionals (eg, social workers, speech-language pathologists) could have enriched topics pertaining to the alignment of virtual care with these practice disciplines. Furthermore, this study focuses on HCPs' perspectives only. Patient and family caregiver perspectives were also collected in a related study and will be reported separately, which will further inform understanding of the implementation and impact of hybrid stroke rehabilitation. Thus, while our study has begun to elucidate issues related to this topic, there may be additional nuances that were not captured.

### Conclusion

This study adds insights into the implementation considerations of the HOSTP program, one of the few outpatient hybrid programs established for the population with stroke using the CFIR framework. The adaptability and relative advantages of the HOSTP, as well as its use and value, were predominantly driven by how well it met patient needs, alongside HCPs’ beliefs, self-efficacy with virtual care, and their involvement in program planning and implementation. These findings suggest that therapy type and specific session goals must guide the choice of delivery medium. Not all rehabilitation translates safely to a video interface, and clinicians need to exercise judgment in that triage. Caution should be exercised to ensure that future virtual care initiatives work to expand access, rather than create new disparities. This includes leveraging hybrid models that afford the flexibility and patient-centeredness of virtual modalities, while ensuring it does not replace in-person care for those that need it.

## Supplementary material

10.2196/83081 Multimedia Appendix 1 Health care providers’ interview guide.

10.2196/83081 Checklist 1 SRQR checklist.

10.2196/83081 Checklist 2 Completed COREQ 32-item checklist.

## Abbreviations

CFIR: Consolidated Framework for Implementation Research
COREQ: Consolidated Criteria for Reporting Qualitative Research
HCP: health care provider
HOSTP: hybrid outpatient stroke telerehabilitation program
OT: occupational therapist
OTA: occupational therapist assistant
PT: physiotherapist
PTA: physiotherapist assistant
SRQR: Standards for Reporting Qualitative Research
