# Supplementary material for: Health Care Providers’ Perspectives on a Hybrid Outpatient Stroke Telerehabilitation Program: Qualitative Implementation Study
Source: JMIR Rehabil Assist Technol. 2026 Jun 15;13:e83081. doi: 10.2196/83081 (PMC13268260; doi:10.2196/83081)
Supplement: Multimedia Appendix 1 [file rehab-v13-e83081-s001.pdf]

Sunnybrook
ST. JOHN'S REHAB

St. John's Rehab
285 Cummer Avenue
Toronto, ON Canada M2M 2G1
Tel: 416.226.6780 | Fax: 416.226.6265
www.sunnybrook.ca/stjohnsrehab

Health Care Professional Interview Guide

"Thank you for participating in this interview. We have included this interview guide for you to know the type of questions to expect and allow you to think about your responses before the interview. From this interview, we hope to gain a better understanding of your experience with preparing and delivering care as part of the hybrid outpatient stroke rehab program at St. John's Rehab. Hybrid refers to a mix of virtual and in-person services, but some patients may only receive virtual care within this program. The questions below will guide our conversation but may vary slightly based on how the conversation unfolds. Please feel free to share as much or little as you are comfortable with during the interview."

1. To begin, could you tell me about your clinical role and work history?
    a. How long have you worked in your role at St. John's Rehab?
    b. What is your current role in the hybrid outpatient stroke rehab program?
    c. Do you only provide virtual services or a mix of virtual and in-person?
    d. Did you have any experience with virtual care prior to this program?
    e. What were your expectations about virtual care?
        i. What concerns did you have? What were you looking forward to?

2. What has your experience been working in the hybrid outpatient stroke rehab program?
    a. How did the actual experience of working in the hybrid outpatient program compare to your expectations?
    b. How prepared did you feel ito provide virtual care when you started this form of care? How do you feel now?
    c. What has been going well? What has helped things go well?
    d. What could be improved? What has posed a challenge?

3. Thinking about your experience with hybrid outpatient stroke rehab, what do you feel is going well? (Can relate to assessment, treatment, discharge planning):
    a. Why do you think it has been going well?
    b. What made these aspects successful?
    c. How do you think we can ensure the continuation of these positive aspects?

4. Similarly, thinking about your experience with hybrid outpatient stroke rehab, what do you feel could be improved? (Can relate to assessment, treatment, discharge planning):
    a. Why do you think these aspects require improvement?
    b. What has posed a challenge?
    c. What do you think is needed for these improvements to become a reality? What would pose a challenge to making them a reality?

St. John's Rehab
285 Cummer Avenue
Toronto, ON Canada M2M 2G1
Tel: 416.226.6780 | Fax: 416.226.6265
www.sunnybrook.ca/stjohnsrehab

5. How do you feel about the extent and quality of care delivered by the hybrid outpatient stroke rehab program?
   a. What are your thoughts on the hybrid nature of the program? (i.e. some in person and some virtual care)
   b. What type of care and therapies do you feel virtual care lends itself to? What types are challenging?
   c. Are there any additional treatments or services you would like to deliver if resources allowed?
   d. Is there anything currently included in the program that you think could be removed? Why?
   e. How do you involve patients and their families/caregiver in care planning when working in a virtual context? (Can relate to assessment, treatment, discharge planning)
   f. Do you feel COVID-19 has impacted the delivery of virtual care within this program?

6. What role do you think hybrid outpatient rehab play in stroke patients' recovery?
   a. What role does outpatient therapy play broadly? What does the virtual aspect of outpatient rehab add or take away?
   b. What can be done to make virtual care more impactful for stroke patients?

7. What has your experience been with discharge planning in the virtual setting?
   a. How does discharge from hybrid outpatient rehab differ from in-person outpatient rehab?
   b. How comfortable and prepared do you feel to discharge plan with patients and families in the virtual context?
   c. What added supports do you need, if any?

8. If resources were not a factor, what would your ideal vision of the hybrid outpatient stroke rehab program be?
   a. What staffing needs are required?
   b. What equipment or services would you include?
   c. What kind of patients would benefit the most in the hybrid outpatient stroke program? Other types of stroke patients?
   d. How would the larger system interact with this program? (in-patient acute care, community, non-virtual or other virtual programs)
   e. How would you measure the success of the new program?

"Thank you for sharing your time and experiences with me. This is the end of our interview questions. Are there other experiences with working in the hybrid outpatient stroke program at St. John's Rehab you'd like to share?" *[If yes, let HCP discuss and if no, reiterate gratitude for their time and participation]*
